# Regulation of Rhizobial Nodulation Genes by Flavonoid‐Independent NodD Supports Nitrogen‐Fixing Symbioses With Legumes

**DOI:** 10.1111/1462-2920.70014

**Published:** 2025-01-25

**Authors:** Timothy L. Haskett, Louise Cooke, Patrick Green, Philip S. Poole

**Affiliations:** ^1^ Department of Biology University of Oxford Oxford UK

**Keywords:** endomycorrhizal fungi, endosymbionts, gene expression/regulation, microbe:higher organism interactions, soil microbes, symbionts

## Abstract

Rhizobia and legumes form a symbiotic relationship resulting in the formation of root structures known as nodules, where bacteria fix nitrogen. Legumes release flavonoids that are detected by the rhizobial nodulation (Nod) protein NodD, initiating the transcriptional activation of nod genes and subsequent synthesis of Nod Factors (NFs). NFs then induce various legume responses essential for this symbiosis. Although evidence suggests differential regulation of *nodD* expression and NF biosynthesis during symbiosis, the necessity of this regulation for the formation of nitrogen‐fixing nodules remains uncertain. Here, we demonstrate that deletion of the *Rlv*3841 NodD regulatory domain results in a constitutively active protein (NodD_FI_) capable of activating NF biosynthesis gene expression without the presence of flavonoids. Optimised constitutive expression of *nodD*_*FI*_ or *nodD3* in *nodD* null mutants led to wild‐type levels of nodulation and nitrogen fixation in pea and *M. truncatula* , respectively, indicating that flavonoid‐regulated *nodD* expression is not essential for supporting symbiosis. These findings illustrate that transcriptional control of flavonoid‐independent NodD regulators can be employed to drive NF biosynthesis, which holds potential for engineering symbiosis between rhizobia and cereals equipped with reconstituted NF receptors.

## Graphical abstract

Legumes release flavonoids into the soil, which are detected by the rhizobial NodD protein, initiating the transcription of *nod* genes for Nod Factors (NF) synthesis. We found that removing the NodD regulatory domain results in a constantly active protein (NodDFI), which independently activates NF biosynthesis genes without flavonoids.

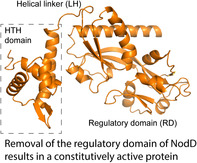

## 1 Introduction

Legumes possess the remarkable ability to form symbioses with rhizobial bacteria. Housed within root nodule structures, rhizobia differentiate into bacteroids and fix atmospheric dinitrogen (N_2_) into NH_3_, which is released for assimilation by the plant (Poole, Ramachandran, and Terpolilli 2018). In return for a source of available nitrogen (N), plants provide bacteroids with carbon in the form of dicarboxylates (Mitsch et al. 2018). These rhizobia‐legume root nodule symbioses (RNS) are critical to future prospects of sustainable agriculture because legume crops can be grown with little to no supplementation with chemically synthesised N fertilisers that are costly to produce, consuming approximately 1% of the world's energy production, and when applied are harmful to the environment, facilitating N leaching (Huang, Ju, and Yang 2017), soil acidification (Tian and Niu 2015), and evolution of nitrous oxide, a potent greenhouse gas (Shcherbak, Millar, and Robertson 2014).

Rhizobia infect legume nodules by either Nod factor (NF)‐dependent or independent entry, with the former strategy being the most common (Masson‐Boivin et al. 2009). During the establishment of NF‐dependent RNS, legumes exude flavonoids, and in some cases, methoxychalcones, aldonic acids, and betaines, which are perceived by rhizobial LysR family NodD proteins. Upon sensing an inducer, NodD drives expression of strain‐specific suites of *nod* genes that are required for synthesis of lipo‐chitooligosaccharides termed NFs. Nod factors are secreted from the rhizobia and bind compatible plant LysM receptor complexes (Krönauer and Radutoiu 2021), triggering symbiosis signalling and downstream responses such as nodule organogenesis and intracellular infection (Yang et al. 2022; Jones et al. 2007; Murray 2011). Multiple isoforms of NodD have been identified in rhizobia (Spaink et al. 1987). NodD in *Rhizobium leguminosarum* bv. *viciae* (*Rlv*) 3841 is activated by various flavonoids (Peck et al. 2013). In contrast, *Sinorhizobium meliloti* (*Sm*) 1021 encodes three copies of NodD (Barnett et al. 2001). NodD1 is activated by luteolin (Peck et al. 2013) and by 4,4′‐dihydroxy‐2′‐methoxychalcone (MCh) (Hartwig et al. 1990), whereas NodD2 is activated by MCh, trigonelline, and stachydrine (Phillips, Joseph, and Maxwell 1992), and NodD3 is a “flavonoid‐independent” (FI) activator that is involved in a self‐amplifying positive regulatory circuit with SyrM (Swanson, Mulligan, and Long 1993; Barnett and Long 2015). The stringency of rhizobial NodD proteins to perceive legume‐derived inducers, paired with the stringency of legume LysM receptor complexes to perceive rhizobial NFs, imparts two stages of host specificity on symbiotic establishment (Wang et al. 2012).

**FIGURE 1:**
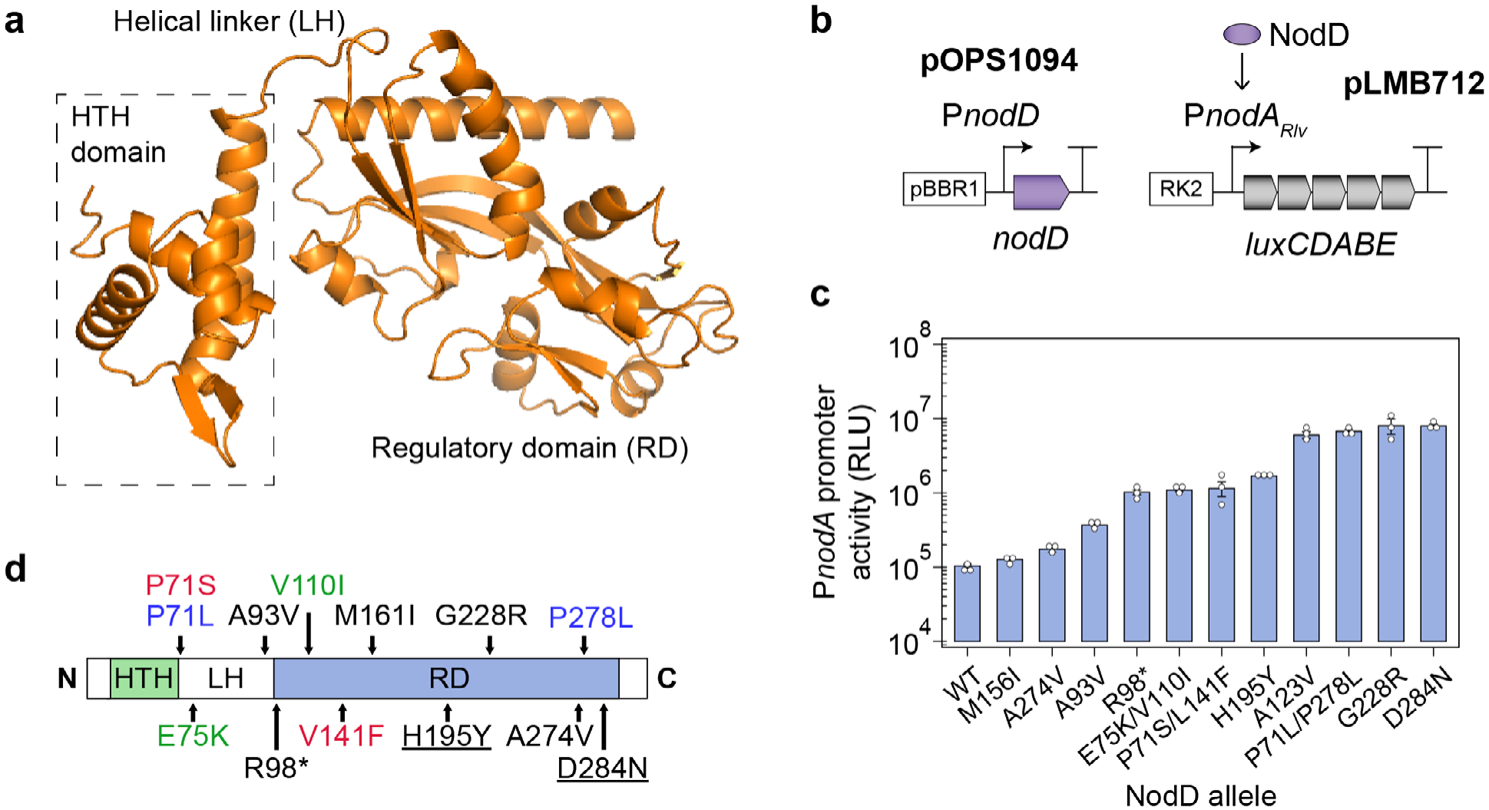
Class IV mutations in NodD_*Rlv*_ generated by random mutagenesis. (a) Tertiary structure of NodD from *Rhizobium leguminosarum* bv. *viceae* 3841 (*Rlv*) modelled using AlphaFold. (b) Genetic schematic of plasmids used for Lux‐based screening of mutagenised NodD proteins for flavonoid‐independent (FI) activity. (c) Bioluminescence readout for activation of P*nodA*_*Rlv*_‐*luxCDABE* cassette in *Rlv* by wild‐type or mutant NodD proteins. Error bars represent one SEM (*n =* 3). (d) Primary structure of NodD_*Rlv*_ showing substitution mutations that imparted FI activity. Matching coloured mutations indicate where two mutations were found in a single allele. Underlined mutations were consistent with those identified in previous studies (Peck et al. 2013; Burn, Rossen, and Johnston 1987; Burn et al. 1989).

Although the structure of NodD and the precise mechanism of action by which NodD activates expression of NF biosynthesis genes have yet to be functionally resolved, bioinformatic analyses indicate that NodD proteins comprise two functional domains, a N‐terminal winged helix‐turn‐helix (HTH) domain and a C‐terminal regulatory domain (RD) that presumably acts as a site for flavonoid interaction (Figure 1a) (Peck et al. 2013; Kostiuk et al. 2013). The two domains are joined by a linker helix (LH). Given the structural conservation of flavonoid‐dependent NodD proteins across species and genera (Schlaman, Okker, and Lugtenberg 1992) and the evidence that NodD proteins can function to activate promoters across species (Peck, Fisher, and Long 2006; Kamboj et al. 2010), it seems likely that the mechanism of flavonoid‐dependent activation would be conserved. Functional studies suggest that NodD forms a cyclically symmetric homodimer or V‐shaped homotetramer that binds two DNA regions of the “*nod*‐box”, termed the distal (D)‐half and proximal (P)‐half on the same face of the DNA helix. When incubated with the cognate flavonoid, the complex activates downstream NF gene expression by inducing a bend in the DNA necessary for RNAP recruitment (Chen et al. 2005; Fisher and Long 1993; Feng et al. 2003; Fisher et al. 1988). Interestingly, various single point mutations introduced along the length of the NodD1 proteins can produce “class IV” mutant proteins capable of activating expression of NF biosynthesis genes in the absence of flavonoids (Peck et al. 2013; Burn, Rossen, and Johnston 1987; Burn et al. 1989; Vinardell et al. 2004). Similarly, mutation of the D‐half P*nodA nod*‐box prevents binding by natural isoforms of NodD, which permits transcriptional activation of the downstream NF biosynthesis genes in the absence of the flavonoid‐inducer, albeit with reduced affinity for activation (Feng et al. 2003). Insertion of 1–2 bp of DNA into both the D‐half and P‐half *nod* has an even more profound effect, inducing a sharp bend in the P*nodA* promoter, which drove constitutive expression of the downstream genes entirely independently of NodD (Chen et al. 2005).

Evidence suggests that *nodD* expression and NF biosynthesis may be differentially regulated throughout infection and, in some cases, during the later stages of symbiosis. However, it remains unclear as to whether this regulation is critical to the formation of N‐fixing nodules. In *Rhizobium‐*pea RNS, *nodD* expression, NodD binding to *nod‐*boxes, expression of *nod* genes, and Nod protein concentration are markedly reduced in bacteroids compared to undifferentiated bacteria, suggesting that there is repression of NF biosynthesis in this state (Schlaman, Lugtenberg, and Okker 1992; Schlaman et al. 1991). A NF biosynthesis gene repressor, NolR, was identified in *Sinorhizobium* and *Rhizobium* that competes for binding with RNAP at the overlapping *nodD* and *nodABCIJ* promoters (Kiss et al. 1998; Kondorosi et al. 1989, 1991). Deletion of NolR in *S. meliloti* AK631 abolished repression of *nod* genes and resulted in delayed nodulation of *Medicago sativa* (Cren, Kondorosi, and Kondorosi 1995), indicating that this mode of regulation is critical for optimal RNS. In contrast, *nolR* mutants of *S. medicae* WSM419 had 2.3‐fold greater competitiveness for nodulation of *M. truncatula* A17 compared to the wild type and showed significantly enhanced nodule development on *M. tricycla* (Sugawara and Sadowsky 2014). Select representatives of the *Bradyrhizobium* genus encode two *nodD* genes: *nodD1*, which functions as a negatively autoregulated FI transcriptional activator of the NF biosynthesis genes (Göttfert et al. 1992; Kosslak et al. 1987), and *nodD2*, which is a transcriptional repressor of the *nodABCIJ* and *nolA* genes transcriptionally controlled by *nolA* itself (Gillette and Elkan 1996; Garcia et al. 1996). *Mesorhizobium loti* also encodes two NodD proteins, but these synergistically direct NF biosynthesis at different stages of symbiosis with *Lotus japonicus* (Kelly et al. 2018). NodD1 is primarily responsible for the induction of *nod* genes within root hair infection threads, whereas NodD2 is primarily responsible for *nod* gene induction in the rhizosphere and nodules. Lastly, it has been demonstrated that the constitutive expression of *Sm nodD3* from a plasmid‐borne P*lac* promoter drives the nodulation of flavonoid‐deficient mutants of *M. truncatula*, although it was not determined whether the nodules formed were fixing N_2_ (Zhang et al. 2009).

Because perception of legume‐exuded inducers by NodD leading to NF biosynthesis represents the earliest stage of signalling by rhizobia in RNS (Wang et al. 2012), establishing transcriptional control of FI NodD proteins such as *Sinorhizobium meliloti* NodD3 or mutated NodD1 proteins (Peck et al. 2013; Burn, Rossen, and Johnston 1987; Burn et al. 1989) could be used to stimulate NF biosynthesis with legumes outside of the natural host range, or even with non‐legumes such as cereals that do not produce compatible flavonoid profiles. The latter would represent a critical step in the engineering of RNS into cereals (Rogers and Oldroyd 2014; Oldroyd and Dixon 2014), as it could be used to activate engineered NF receptor complexes (Krönauer and Radutoiu 2021; Gysel et al. 2021; Bozsoki et al. 2020). FI NodD proteins could even be placed under transcriptional control by natural or engineered non‐legume signals to introduce stringency to the interaction and prevent NF signalling with non‐target crops. This strategy has already been used to control N‐fixation and ammonia excretion by *Azorhizobium caulinodans* colonising engineered rhizopine‐producing (*RhiP*) barley plants (Haskett et al. 2022a, 2022b; Geddes et al. 2019).

In this study, we demonstrate that removal of the *Rlv* 3841 NodD (here termed NodD_*Rlv*_) regulatory domain yields a constitutively active protein comprised of the HTH‐LH domains that alone drive expression of the NF biosynthesis genes in the absence of a cognate flavonoid inducer, albeit with reduced affinity for activation compared to the wild‐type NodD protein. Both the truncated *nodD*_*Rlv*_, herein termed *nodD*_*FI*_ (flavonoid‐independent), and *nodD3* from *Sm* CL150 (here termed *nodD3*_*Sm*_) could be expressed from an inducible promoter in *nodD* null mutants of the parent strains, leading to tunable control of the P*nodA* promoters upstream of the core *nodABCIJ* genes. Optimised expression of *nodD*_*FI*_ or *nodD3*_*Sm*_ from constitutive promoters in *nodD* null mutants of the parent strains resulted in wild‐type levels of nodulation and N‐fixation on pea and *M. truncatula*, respectively, demonstrating that *nodD* expression does not need to be differentially regulated by flavonoids throughout symbiosis to support infection of legume nodules and N‐fixation. These findings provide new insights into the mechanism by which the NodD protein functions and demonstrate that transcriptional control of FI NodD regulators can be effectively utilised to control NF biosynthesis, which will be critical for engineering RNS between rhizobia and non‐legumes.

## 2 Results

### 2.1 Generation of Class IV NodD Mutations by Random Mutagenesis

NodD proteins are highly conserved in legume‐nodulating rhizobia and are comprised of a DNA‐binding HTH domain, a LH, and an RD (Figure 1a), the latter of which acts as a site for interaction with flavonoid inducers that modulate the protein's ability to activate expression from *nod* gene promoters. Several “class IV” mutations have been characterised in rhizobial NodD proteins that permit NodD to activate *nod* promoters in the absence of flavonoids (Peck et al. 2013; Burn, Rossen, and Johnston 1987; Burn et al. 1989; Vinardell et al. 2004). We aimed to generate a collection of class IV mutant alleles for the *Rlv*3841 *nodD*_*Rlv*_ gene. To achieve this, *nodD*_*Rlv*_ and the upstream promoter were cloned into plasmid pBBR1‐MCS2 in opposite orientation to the resident P*lac* promoter, producing pOPS1094, which was subjected to hydroxylamine mutagenesis and transformed into *E. coli* . A library of approximately 7500 mutagenised plasmid transformants was pooled and mass conjugated with *Rlv*3841 harbouring the P*nodA*_*Rlv*_‐*luxCDABE* reporter plasmid pLMB712 (Figure 1b). Single colonies of plasmid transconjugants were selected on TY agar supplemented with tetracycline, kanamycin, and gentamycin, without the cognate flavonoid hesperitin. Using *Rlv*3841 carrying unmutagenised pOPS1094 and pLMB712 as a negative control, we screened single colonies for activation of the P*nodA*_*Rlv*_‐*lux* fusion by mutagenised NodD_*Rlv*_ using a NightOWL photon counting camera system. Plasmids were extracted from twenty bioluminescent rhizobial colonies, transformed into *E. coli* and conjugated into *Rlv*3841 carrying pLMB712 to confirm that P*nodA*_*Rlv*_‐*lux* activation was due to class IV NodD_*Rlv*_ activity rather than a mutation in the *lux* cassette (Figure 1c). Finally, the *nodD*_*Rlv*_ mutation(s) were characterised by Sanger sequencing on both DNA strands, revealing that 10 of the alleles were unique (Figure 1d). Two of the *nodD*_*Rlv*_ alleles contained two substitutions, one in the LH and the other in the RD, whereas the remaining mutations existed in the RD of the protein. Two of the single substitutions, H195Y and D284N, have been previously described (Peck et al. 2013; Burn et al. 1989), whereas the remaining 8 substitutions have not previously been identified (Peck et al. 2013; Burn et al. 1989; Vinardell et al. 2004). Interestingly, one of the mutations in our random mutagenesis experiments substituted an arginine residue for a stop codon at the C‐terminus of the LH domain (R98*), which would produce a truncated protein only containing the HTH and LH domains (Figure 1a,c). This suggested that the RD may act as a repressor of the HTH‐LH domains, which could alone activate expression of LCO biosynthesis genes.

### 2.2 The NodD HTH‐LH Domains Exhibit Flavonoid‐Independent Activity

**FIGURE 2:**
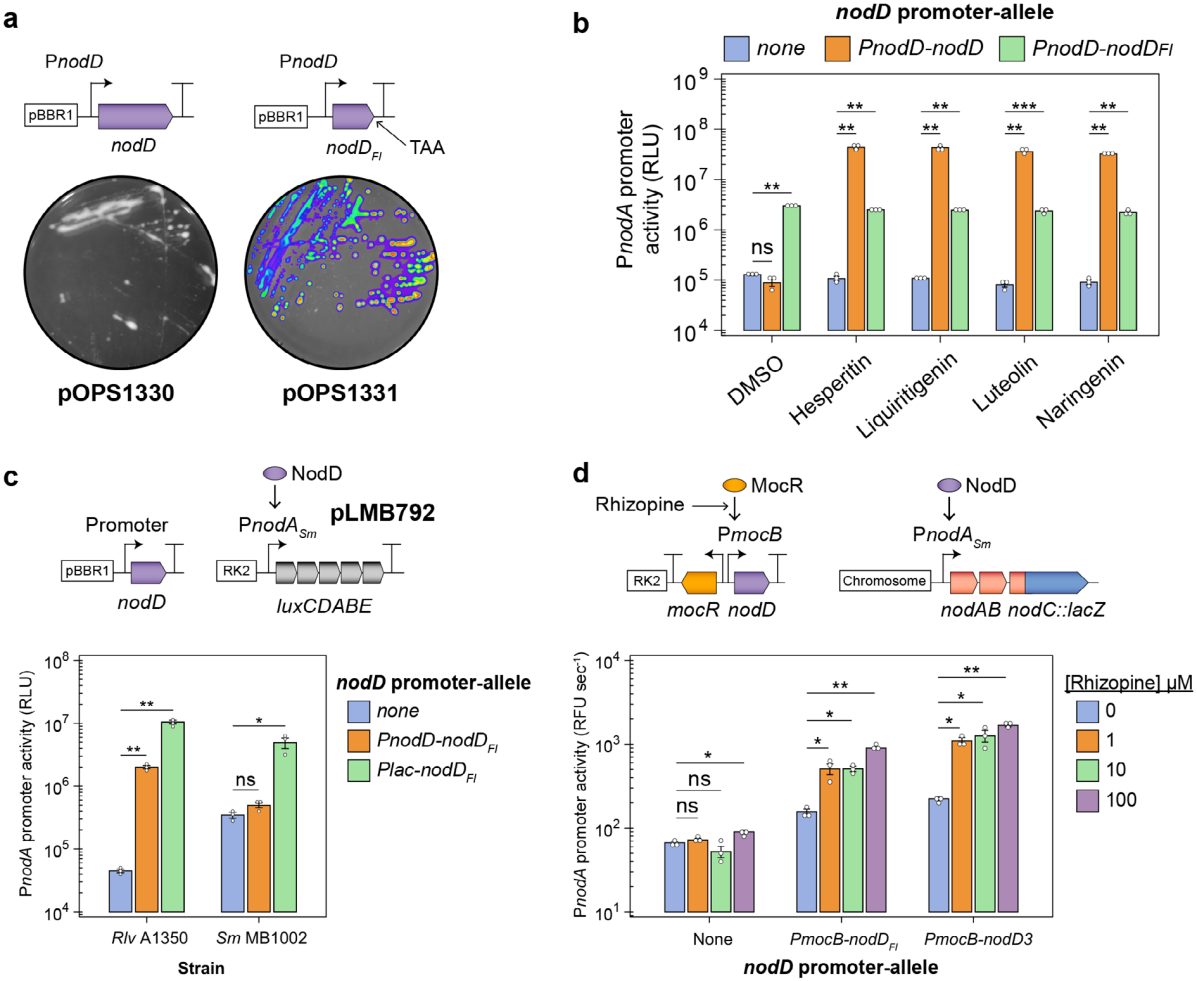
The NodD_*Rlv*_ HTH‐LH domains activate P*nodA* expression independently of flavonoids. (a,b) Flavonoid‐independent (FI) activation of the P*nodA*_*Rlv*_ *‐luxCDABE* reporter cassette (plasmid pLMB712) in *Rhizobium leguminosarum* bv. *viceae* (*Rlv*) 3841 by *nodD*_*FI*_. The *nodD*_*FI*_ gene is comprised of the first 294 bp of *nodD*_*Rlv*_ with an introduced stop codon (TAA). The scale in a is from 500 to 8500 counts per second (cps). (c) Flavonoid‐independent (FI) activation of the P*nodA*_*Sm*_ *‐luxCDABE* reporter cassette (plasmid pLMB792) in *nod* null mutants of *Rlv* (strain A1350) and *Sinorhizobium meliloti* (*Sm*, strain MB1002) by *nodD*_*FI*_. (d) Tunable rhizopine‐inducible *nodD*_*FI*_ and *nodD3* expression in a *Sm nodD* null mutant chromosomal *nodC::LacZ* fusion (strain MB1003). Error bars represent one SEM (*n =* 3). Independent two‐tailed students t‐tests were used to compare means. Not significant (ns *p* > 0.05), *p < 0.05, **p < 0.01, ***p < 0.01. Bacteria in reporter assays were induced for 24 h prior to measurement.

To further explore whether the NodD_*Rlv*_ RD was dispensable for activity, the native *nodD*_*Rlv*_ promoter and the first 294 bp (98 amino acids) of the coding sequence were amplified from wild‐type *Rlv*3841 genomic DNA with primers that substituted R98 for a stop codon (TAA), and the amplicon was cloned into pOGG024, creating pOPS1331. This plasmid and a second pOGG024‐derived plasmid containing the native *nodD*_*Rlv*_ gene and promoter (pOPS1330) were each mobilised into a *nod*‐Tn*5* mutant of *Rlv*3841 (A1350) harbouring the P*nodA*_*Rlv*_‐*lux* reporter plasmid pLMB712 (Figure 1b). *Rlv*A1350 carrying the truncated *nod*_*Rlv*_ allele, herein termed *nodD*_*FI*_ (flavonoid‐independent), exhibited clear bioluminescence compared to the control strain in the absence of flavonoids (Figure 2a). The activities of NodD_*Rlv*_ compared to NodD_FI_ were also tested when flavonoids were added to the growth media in bioluminescence assays (Figure 2b). As expected, the addition of either 5 μM naringenin, liquitigenin, hesperitin or luteolin activated the native NodD_*Rlv*_ allele in *Rlv*A1350 whereas NodD_FI_ was unaffected by the addition of flavonoids to the growth media, and was constitutively active, albeit with reduced affinity for activation of P*nodA*_*Rlv*_‐*lux* compared to the activated wild‐type allele.

We next tested whether NodD_FI_ could activate a *Sinorhizobium meliloti* P*nodA*_*Sm*_‐*lux* reporter carried on plasmid pLMB792 in strain MB1002 (a *nodD1/nodD2/nodD3* mutant of *Sm*CL150) by introducing pOPS1331 (P*nodD‐nodD*_*FI*_) and the empty control vector pOGG024. We found a small, but statistically insignificant increase in bioluminescence for the strain carrying P*nodD‐nodD*_*FI*_ compared to the control strain, indicating that NodD_FI_ may activate P*nodA*_*Sm*_‐*lux* in the *Sm*CL150 background with low affinity (Figure 2c). To increase *nodD*_*FI*_ expression in this background, we subcloned *nodD*_*FI*_ under the control of the stronger constitutive P*lac* promoter in pOGG024 and mobilised the resulting plasmid pOPS1368 into *Sm*CL150 carrying pLMB792. The resulting strain showed strong bioluminescence compared to the control, confirming that *nodD*_*FI*_ could activate P*nodA*_*Sm*_‐*lux* in the *Sm* background (Figure 2c).

### 2.3 Transcriptional Control of Flavonoid‐Independent *nodD* Regulators

Establishing transcriptional control of NodD_FI_ or similar regulators such as NodD3 from *Sm* (here termed NodD3_*Sm*_) could be exploited to permit plant signal‐dependent activation of rhizobial NF biosynthesis in bacteria colonising non‐legumes that do not produce adequate concentrations of cognate flavonoids required for native NodD activation. We had previously engineered barley that excreted the signalling molecule rhizopine, which could be perceived by bacteria carrying rhizopine biosensors in the rhizosphere (Haskett et al. 2022a). Here, we tested whether rhizopine control of *nodD*_*FI*_ and *nodD3*_*Sm*_ could be used to control activation of NF biosynthesis genes by cloning these alleles downstream of the rhizopine‐inducible promoter (P*mocB*) in our biosensor plasmid pOPS1052, forming plasmids pOPS1090 and pOPS1091, respectively. When mobilised into *Sm*MB1003 (a *nodD1/nodD2/nodD3* mutant of *Sm*CL150 harbouring a chromosomal *nodC‐lacZ* reporter fusion), the addition of SI to both cultures stimulated tunable activation of the upstream P*nodA*_*sm*_ promoter, with NodD3_*Sm*_ exhibiting a stronger affinity for activation (Figure 2d). This result indicated that plant signal‐dependent transcriptional control over FI NodD regulators could be used to drive expression of rhizobial NF biosynthesis genes.

### 2.4 Constitutive Expression of *nodD*_*FI*_ Supports Nitrogen‐Fixing Symbiosis With Pea

Rhizobial LCOs matched to compatible legume LysM receptors must be produced at defined concentrations to establish functional N‐fixing symbioses between rhizobia and legumes. Considering this, we tested whether NF biosynthesis driven by constitutive expression of *nodD*_*FI*_ in *Rlv*A1350 would permit nitrogen‐fixing symbiosis with pea. For this experiment, *nodD*_*FI*_ was subcloned onto the stable broad host‐range plasmid pOGG093 under transcriptional control by one of three constitutive promoters, P*rpoD*, PJ23115, or PJ23106, ordered by increasing strength (Figure A1). The resulting plasmids, pOPS2023, pOPS2024, and pOPS1538, respectively, were mobilised into *Rlv*A1350 harbouring a chromosomal GFP cassette (*Rlv*A1350‐GFP) to permit cell tracking.

**FIGURE 3:**
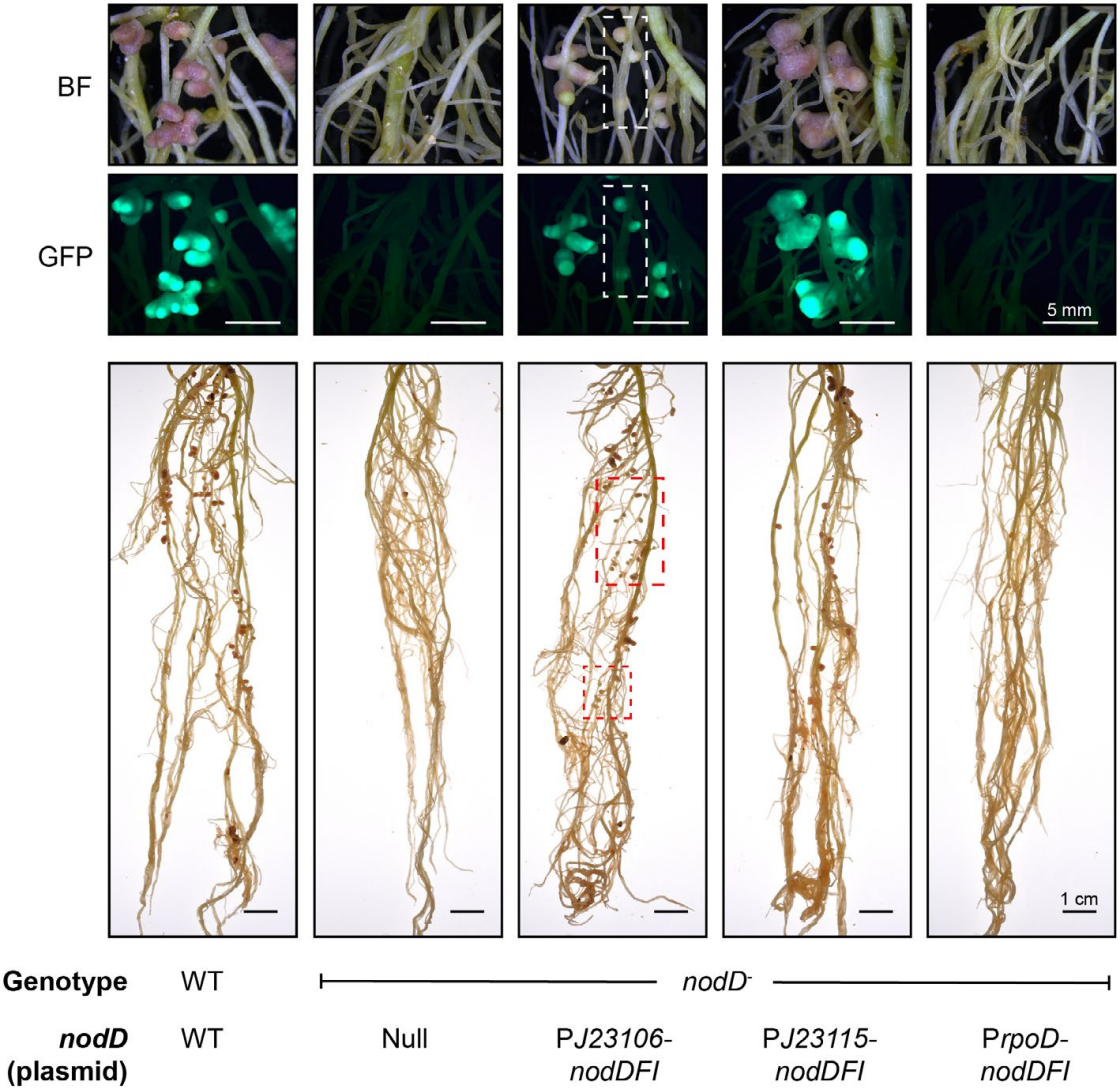
Constitutive expression of *nodD*_*FI*_ in a *nod* null mutant of *Rlv*3841 drives nodulation of pea. Images were taken for whole root systems of N‐free‐grown pea plants harvested 28 days post inoculation with wild‐type (WT) *Rhizobium leguminosarum* bv. *viceae* (*Rlv*) 3841 or *nod‐* mutants (A1350) carrying *nodD*_*FI*_ expressed from constitutive promoters varying in their strength (see Figure A1). All strains were marked with a constitutively expressed GFP gene for tracking in nodules. Dashed boxes highlight white nodules. BF, bright field; GFP, green fluorescent protein.

**FIGURE 4:**
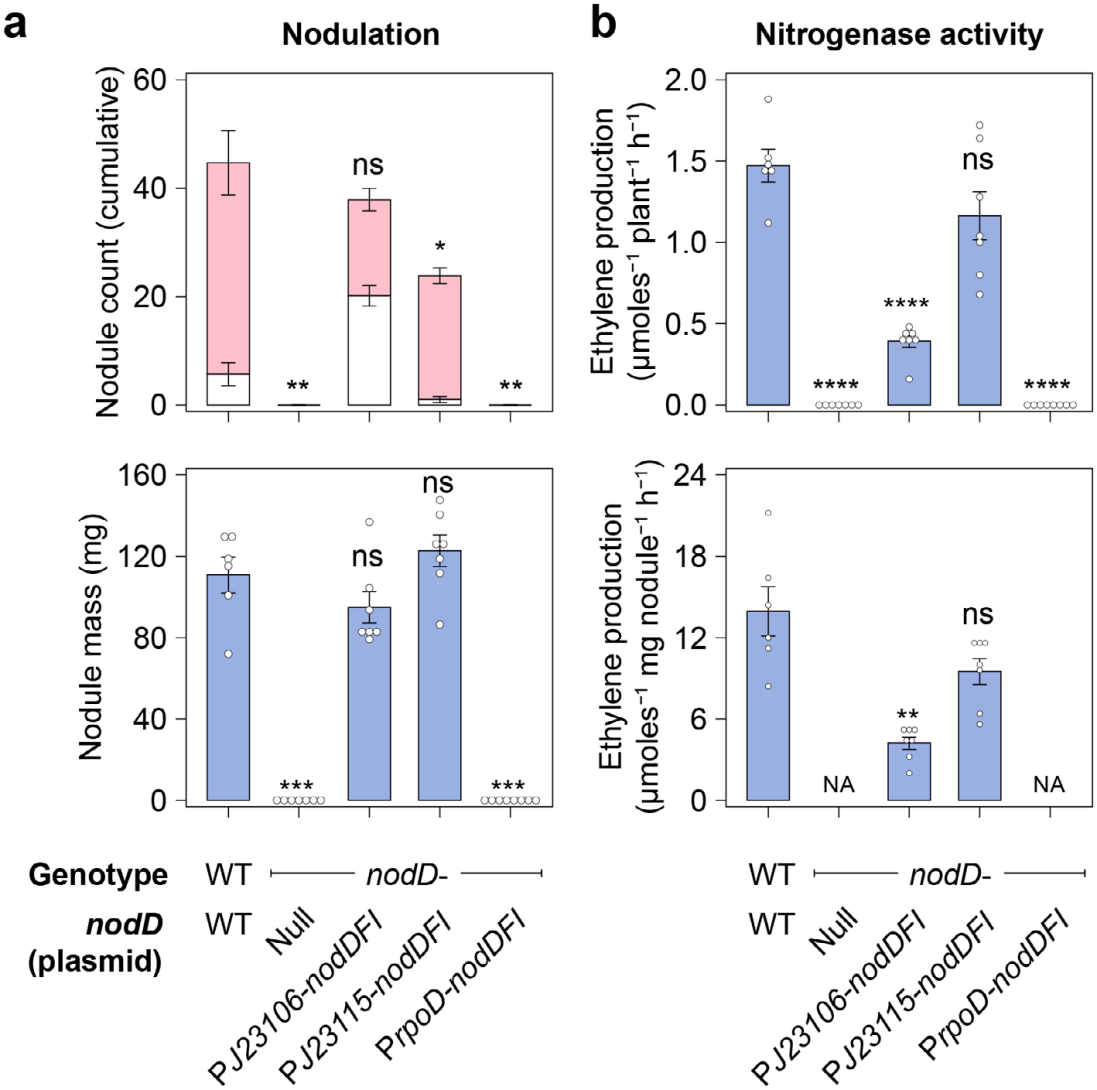
Constitutive expression of *nodD*_*FI*_ in a *nod* null mutant of *Rlv*3841 permits functional N_2_‐fixing symbiosis with pea. N‐free‐grown pea plants were harvested 28 days post inoculation with wild‐type (WT) *Rhizobium leguminosarum* bv. *viceae* (*Rlv*) 3841 or *nod‐* mutants (A1350) carrying *nodD*_*FI*_ expressed from constitutive promoters varying in their strength (see Figure A1). All strains were marked with a constitutively expressed GFP gene for tracking in nodules. (a) Nodulation phenotypes give mass and colour‐coded counts for both pink and white nodules. Nodule mass shown is fresh weight. (b) Nitrogenase activity as measured by acetylene reduction assays. Treatments where no nodules formed were not applicable (NA). Error bars represent one SEM. Independent two‐tailed students t‐tests with Bonferroni adjustment were used to compare means. Not significant (ns *p* > 0.05), *p < 0.05, **p < 0.01, ***p < 0.01, ****p < 0.001.

Both the negative control strain *Rlv*A1350‐GFP and the same strain carrying P*rpoD*‐*nodD*_*FI*_ did not form nodules on pea at 28 days post inoculation (dpi) (Figure 3 & Figure 4a). In comparison, *Rlv*A1350‐GFP carrying PJ23115‐*nodD*_*FI*_ or PJ23106‐*nodD*_*FI*_ elicited nodule formation at the same time point. Nodules formed on pea plants inoculated with *Rlv*A1350‐GFP carrying PJ23115‐*nodD*_*FI*_ were pink, indicating the production of leghaemoglobin required for N fixation. Moreover, nitrogenase activity, as measured by acetylene reduction assays (ARA), was no different compared to plants inoculated with the wild‐type control strain *Rlv*3841‐GFP (Figure 4b), indicating that *Rlv*A1350‐GFP carrying PJ23115‐*nodD*_*FI*_ formed a functional nitrogen‐fixing symbiosis with pea. In contrast, approximately 50% of the nodules formed by *Rlv*A1350‐GFP carrying PJ23106‐*nodD*_*FI*_ were white (Figure 3 & Figure 4a), and nitrogenase activity measured in whole plants equated to approximately 33% compared to plants inoculated with a wild‐type control strain (Figure 4b). Both white and pink nodules on plants inoculated with *Rlv*A1350‐GFP carrying PJ23106‐*nodD*_*FI*_ exhibited GFP fluorescence under the stereomicroscope (Figure 3), indicating they were infected with the fluorescently marked inoculant. Furthermore, the mean nodule mass per plant was no different from plants inoculated with the wild‐type control strain, suggesting that the nodules had not been sanctioned (Figure 4a). This data suggested that nodule development on plants inoculated with *Rlv*A1350‐GFP carrying PJ23106‐*nodD*_*FI*_ was delayed compared to the wild‐type control, highlighting that correctly tuned expression of *nodD*_*FI*_ is critical to the development of fully functional nitrogen‐fixing nodules in the *Rhizobium‐*pea symbiosis.

### 2.5 Constitutive Expression of *nodD3* Supports Nitrogen‐Fixing Symbiosis With *Medicago*

**FIGURE 5:**
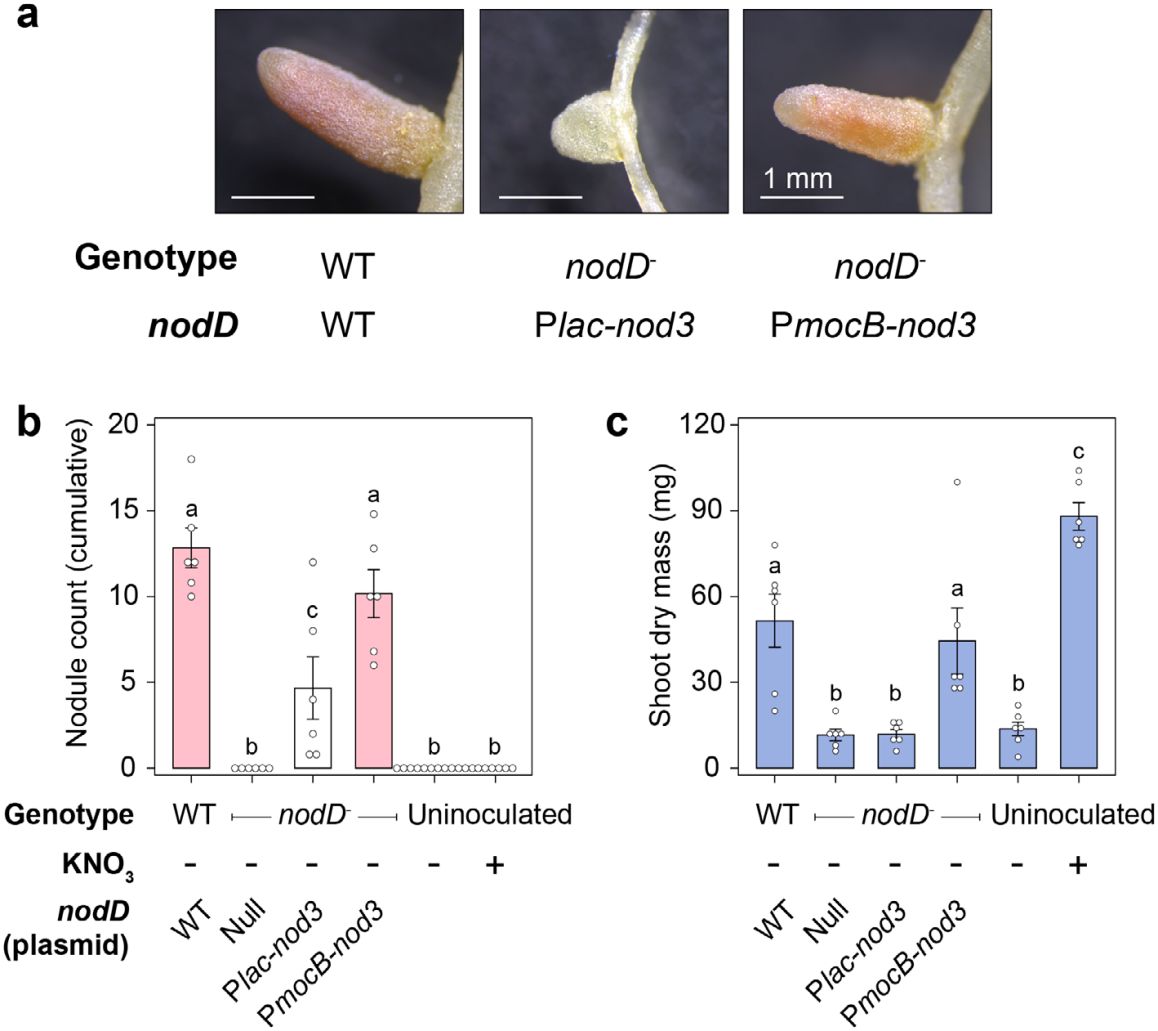
Leaky expression of *nodD3*_*Sm*_ in a *nod* null mutant of *Sm*CL150 permits functional N_2_‐fixing symbiosis with *M. truncatula* . N‐free‐grown *Medicago* plants were harvested 32 days post inoculation with wild‐type (WT) *Sinorhizobium meliloti* (*Sm*) CL150 or *nod‐* mutants (MB1002) carrying *nodD3*_*Sm*_ expressed from inducible promoters varying in their strength (see Figure A2). To keep expression minimal, no inducer was supplied in this experiment. (a,b) Nodulation counts are colour‐coded for pink and white nodules. (b) Under N‐free conditions, shoot dry weight measurements were used to assess the extent of N‐fixation. Error bars represent one SEM. Least Significant Difference (LSD) tests were used to compare means. Matching letters indicate treatments that are not significantly different from one another but are significantly different from non‐matching letters (alpha = 0.05).

We wanted to explore whether constitutive expression of *nodD3*_*Sm*_ could support nitrogen‐fixing symbiosis between *Sm* and *Medicago truncatula* . We utilised two stable, low‐copy, broad host‐range plasmids to maintain low levels of *nodD3*_*Sm*_ expression in the *nodD* null mutant *Sm*MB1002—one where *nodD3*_*Sm*_ was placed under control of the IPTG de‐repressible P*lac* promoter (pOPS1096) and the other where *nodD3*_*Sm*_ was expressed from the rhizopine‐inducible P*mocB* promoter (pOPS1091). It should be noted that while both promoters are inducible, both have significant but low background activity in the absence of the inducer due to the presence of strong ribosome binding sites, with Plac being the stronger of the two promoters in the non‐induced state (Figure A2). *M. truncatula* plants grown in N‐free conditions inoculated with the positive control strain *Sm*CL150 formed pink nodules at 35 dpi, whereas uninoculated plants and those inoculated with the negative control strain *Sm*MB1002 did not form nodules at this time point (Figure 5a,b). Plants inoculated with *Sm*MB1002 carrying P*mocB*‐*nodD3*_*Sm*_ formed a similar number of pink nodules per plant relative to the wild‐type control, whereas plants inoculated with *Sm*MB1002 carrying P*lac*‐*nodD3*_*Sm*_ formed fewer nodules than the wild type (Figure 5b), and these were white and spherical (Figure 5a), suggesting they were not producing leghaemoglobin required for N fixation.

Under N‐free conditions, shoot dry weight measurements of inoculated plants relative to N‐free N‐fed control plants can be used to indirectly quantify nitrogen fixation. We found that the shoot dry weights of plants inoculated with *Sm*MB1002 or the strain carrying P*lac*‐*nodD3*_*Sm*_ were significantly lower than those of plants inoculated with the wild‐type strain *Sm*CL150 and were no different from the uninoculated N‐free control (Figure 5c), indicating these plants did not fix N. In contrast, the shoot dry weights of plants inoculated with *Sm*MB1002 carrying P*mocB*‐*nodD3*_*Sm*_ were no different from those inoculated with the wild‐type control strain and were significantly higher than the uninoculated N‐free control plants, indicating that this strain fixed a comparable amount of N to the wild‐type control. Thus, we conclude that constitutive expression of *nodD3*_*Sm*_ can support nitrogen‐fixing symbiosis between *Sm* and *Medicago truncatula*.

## 3 Discussion

In this study, we have shown that removal of the NodD_*Rlv*_ RD yields a constitutively active protein comprised of the HTH‐LH domains that alone drive expression of the NF biosynthesis genes in the absence of a cognate flavonoid inducer, albeit with reduced affinity for activation compared to the wild‐type protein. Thus, it seems highly likely that the RD acts to inhibit activity of the HTH‐LH domains in the absence of the cognate flavonoid. These results also explain why various single substitution mutations along the length of the domain can readily yield class IV mutant NodD variant proteins (Peck et al. 2013; Burn, Rossen, and Johnston 1987; Burn et al. 1989), because it is likely that they result in loss of function for the RD. However, we cannot rule out the seemingly less likely alternative possibility that removal of the RD could permit FI activation of transcription by preventing NodD_FI_ protein from forming dimers/multimers and (or) interacting with the distal P‐half of the *nod*‐box, both of which were previously shown to result in flavonoid‐independent activation of the P*nodA* promoter (Chen et al. 2005; Fisher and Long 1993; Feng et al. 2003). Further genetic and structural analysis will be necessary to resolve these two possibilities.

Although evidence suggests that *nodD* expression and NF biosynthesis are differentially regulated throughout the stages of symbiotic establishment in multiple rhizobia‐legume symbioses (Schlaman, Lugtenberg, and Okker 1992; Kiss et al. 1998; Kondorosi et al. 1989; Cren, Kondorosi, and Kondorosi 1995; Gillette and Elkan 1996; Garcia et al. 1996), it was previously unclear whether this regulation was critical to the formation and maintenance of effective N‐fixing nodules. While we did not directly measure expression of *nodD*_*FI*_ or *nodD3*_*Sm*_ in *nod* null mutants of *Rlv* 3841 and *Sm* CL150 throughout the various stages of the nodule symbiosis, we observed that tuned expression from promoters that are constitutive in laboratory culture enables formation of fully functional N_2_‐fixing symbioses on pea and *M. truncatula*, respectively. We do, however, acknowledge that many RpoD (σ^70^)‐dependent bacterial genes that are expressed constitutively in free‐living culture and in the rhizosphere are significantly reduced inside of N_2‐_fixing bacteroids, which may also be true for the synthetic promoters tested in this work (Ramachandran et al. 2011; Karunakaran et al. 2009). It is also likely that NF biosynthesis downstream of NodD expression and activity remains subject to regulation by host factors other than NodD, such as NolR (Kiss et al. 1998; Kondorosi et al. 1991; Kim et al. 1989). We also observed here that when FI *nodD* genes were expressed too strongly or weakly, nodulation and (or) nitrogen fixation with pea and *Medicago* were impaired. This is consistent with studies on *S. fredii* HH103, where it was postulated that increased NF biosynthesis due to mutation of *nodD2*, *nolR*, or *syrM* was responsible for impaired symbiosis with *Lotus burttii*, an otherwise compatible host (Acosta‐Jurado et al. 2020, 2019). Overall, these data support the notion that correctly tuned NF biosynthesis is critical throughout RNS. In future work, it will be important to monitor the bacterial transcriptome throughout symbiotic establishment to better understand the full genetic implications of NodD engineering and identify any potentially problematic off‐target effects.

The discovery that constitutive expression of FI *nodD* genes can support N‐fixing symbioses with legumes could be applied to expand the symbiotic host compatibility of rhizobia that do not perceive NF‐inducing signals exuded by target legumes, as was achieved previously by expression of FI *nodD* genes with their native promoters in natural (McIver et al. 1989) or heterologous (Ayala‐García et al. 2022) hosts. Using FI *nod* alleles in this way likely requires that NF production is tuned appropriately and that the correct suite of *nod* genes is present in the bacteria. The *nod* gene complement could exist naturally or could be artificially introduced by conjugal transfer of symbiotic plasmids (Ruiz‐Sainz, Jiminez‐Diaz, and Beringer 1984), ICEs (Haskett 2018; Hill et al. 2021; Sullivan and Ronson 1998), or by more targeted genetic engineering approaches. FI *nodD* genes could also be used to control NF biosynthesis in non‐symbiotic bacteria carrying symbiotic plasmids (Brom et al. 1988), or engineered *nod* clusters akin to nitrogenase (*nif*) clusters built for the transfer of nitrogen fixation capacity in free‐living bacteria (Ryu et al. 2020; Temme, Zhao, and Voigt 2012). Constitutive transcriptional control of FI NodD proteins could also be used to drive NF production by bacteria and activate engineered NF receptors in cereals (Krönauer and Radutoiu 2021; Bozsoki et al. 2020). However, control circuitry on NF biosynthesis would ultimately be essential to reduce metabolic burden and prevent silencing of the edited genes. Ideally, FI *nodD* genes would be placed under the control of plant‐derived signals, introducing stringency to the interaction. Such signals could be naturally produced (Galloway et al. 2020) or could be introduced by genetic modification, as is true of engineered rhizopine‐producing (*RhiP*) barley plants (Haskett et al. 2022a; Geddes et al. 2019; Ryu et al. 2020). We have shown that rhizopine signalling circuitry (Haskett et al. 2022b) can be used to drive expression of *nodD*_*FI*_ or *nodD3*_*Sm*_ to stimulate expression of NF biosynthesis genes in free‐living culture. However, *Sm* MB1002 carrying a rhizopine‐inducible *nodD3*_*Sm*_ gene formed N_2_‐fixing nodules when inoculated onto wild‐type *M. truncatula* , indicating that regulation of *nodD3*_*Sm*_ needs to be tighter regulated to permit specific activation by rhizopine‐produicing (*RhiP*) barley plants. Use of tools such as single‐copy mini‐Tn7 integration (Choi and Schweizer 2006), small RNAs (Dutta and Srivastava 2018), or dCas9 (Hawkins et al. 2015) could be used in the future to tighten this leaky expression and establish stringent rhizopine control of NF signalling.

## 4 Materials and Methods

### 4.1 Bacterial Strains and Plasmids

Strains used in this study are described in Table A1. *Escherichia coli* was cultured on LB media (Bertani 1951) at 37°C, whereas rhizobia were cultured on TY (Beringer 1974) or UMS (Poole et al. 1994; Brown and Dilworth 1975) media at 28°C. Plasmids used in this study are described in Table A2 and were constructed using either golden‐gate or HiFi cloning as described in File S1.

### 4.2 Hydroxylamine Mutagenesis and Screening of *nodD* Mutations

*nodD*_*Rlv*_ and its native promoter were initially HiFi cloned into plasmid pBBR1‐MCS2 in opposite orientation to the native P*lac* promoter, producing pOPS1094, and this plasmid was subject to hydroxylamine mutagenesis (Peck et al. 2013; Amberg, Burke, and Strathern 2006). Aliquots of five micrograms of plasmid DNA were mutagenised by incubation at 70°C in 250 uL of a 2 M hydroxylamine HCL solution for 0, 60, 90, or 120 min. Reactions were purified using a NEB Monarch PCR & DNA Cleanup Kit, and five microlitres of the eluted, mutagenised DNA for each treatment were transformed into chemically competent DH5a cells. Transformation of plasmid DNA that was incubated for 120 min with hydroxylamine HCL yielded less than 1% of the number of colonies compared to the control treatment, indicating the DNA was successfully mutagenized. *nodD*_*Rlv*_ and the native promoter were subsequently amplified and sequenced from 10 of these colonies using the M13 primers, and the resulting amplicons were sequenced, revealing that there was approximately 1 transition mutation per 2‐kb plasmid DNA. We washed a total of 7500 transformant colonies from the LB plates to form a *nodD* mutant library, which was frozen at −80°C in 10% DMSO. This library was recovered and tri‐parentally mass‐conjugated with the *E. coli* carrying the helper plasmid pRK2013 and *Rlv*3841 carrying a P*nodA‐luxCDABE* fusion plasmid (pLMB712). Single colonies were screened for induction of the P*nodA‐lux* fusion using a Night‐Owl instrument, and 20 bioluminescent colonies were passaged onto selective media. Plasmids from these colonies were extracted using a Neb Monarch Plasmid Miniprep Kit and triparentally conjugated back into *Rlv*3841 (pLMB712) to confirm *nodD* activity in the absence of flavonoids. The plasmid‐borne mutant *nodD* genes and promoters from each colony were finally Sanger sequenced from each end using the M13 primers to type the mutations present.

### 4.3 Reporter Assays

Bacteria used in reporter assays were inoculated into non‐selective UMS media at OD600nm 0.1 and incubated with the relevant inducer at 28°C for 24 h prior to diluting 1:1 with UMS and measuring fluorescence or luminescence. Relative luminescence units (OD600nm/counts per second, RLU) for *lux* reporter assays were measured using a Promega GloMax multidetection system. Relative fluorescence units (OD600nm/fluorescence intensity, RFU) for GFP and beta‐galactosidase assays were measured with an Omega FLUOstar set at gain 1000. Beta‐galactosidase assays were performed as previously described (Ramsay 2013).

### 4.4 Plant Experiments

All seeds used in this study were surface sterilised by submersion in 70% (v/v) ethanol for 1 min, followed by 5% (v/v) NaOCl for 5 min. After washing 5 times with sterile water, seeds were germinated on water agar in the dark for 3 days prior to sowing, then immediately inoculated with 5 mL of OD600nm 0.1 suspension of inoculant washed from 3‐day‐grown agar slopes. Pea plants (Avola) were sown in boiling tubes containing autoclaved vermiculite and 15 mL of N‐free nutrient solution (Haskett et al. 2021) as previously described. Tubes were supplemented with 10 mL of N‐free nutrient solution weekly and harvested at 28 dpi. Plants were grown in a growth chamber at 25°C with a 16‐h/8‐h day/night cycle. Acetylene reduction assays were performed on single plants as previously described (Haskett et al. 2021), and whole nodules were imaged using a Leica DM4000 B Fluorescence Motorised Microscope.

For *Medicago truncatula* A17, free‐draining pots containing fire sand (Haskett et al. 2021) were flushed with water and autoclaved prior to sowing. Plants were grown 32 days post inoculation prior to harvesting. All N‐free treatments were supplemented with 10 mL of N‐free nutrient solution (Haskett et al. 2021) per week, whereas N‐fed controls were supplemented with the same volume of nutrient solution additionally containing 10 mM KNO_3_. After acetylene reduction assays, nodules were excised and their fresh weights were measured. Roots were then excised from the shoot at the transition zone and dried for 2‐days at 60°C to measure shoot dry weights.

### 4.5 Statistical Data Analysis

Statistical analyses were carried out using the R package Rstatix (Team RC 2021). Details are outlined in the figure captions.

## Author Contributions

**Timothy L. Haskett:** conceptualization, investigation, writing – original draft, methodology, validation, visualization, writing – review and editing, formal analysis, data curation, supervision, funding acquisition. **Louise Cooke:** investigation. **Patrick Green:** investigation. **Philip S. Poole:** conceptualization, investigation, funding acquisition, writing – original draft, methodology, validation, visualization, writing – review and editing, formal analysis, project administration, data curation, supervision, resources.

## Conflicts of Interest

The authors declare no conflicts of interest.

## Supporting information


**Appendix S1.** Supporting Information.

## Data Availability

The data that support the findings of this study are available in the article and its appendix.
