# Supplementary material for: Regulation of Rhizobial Nodulation Genes by Flavonoid‐Independent NodD Supports Nitrogen‐Fixing Symbioses With Legumes
Source: Environ Microbiol. 2025 Jan 25;27(1):e70014. doi: 10.1111/1462-2920.70014 (PMC11771551; doi:10.1111/1462-2920.70014)
Supplement: Supplementary file 1 — Appendix S1. Supporting Information. [file EMI-27-e70014-s001.docx]

**APPENDIX**

**
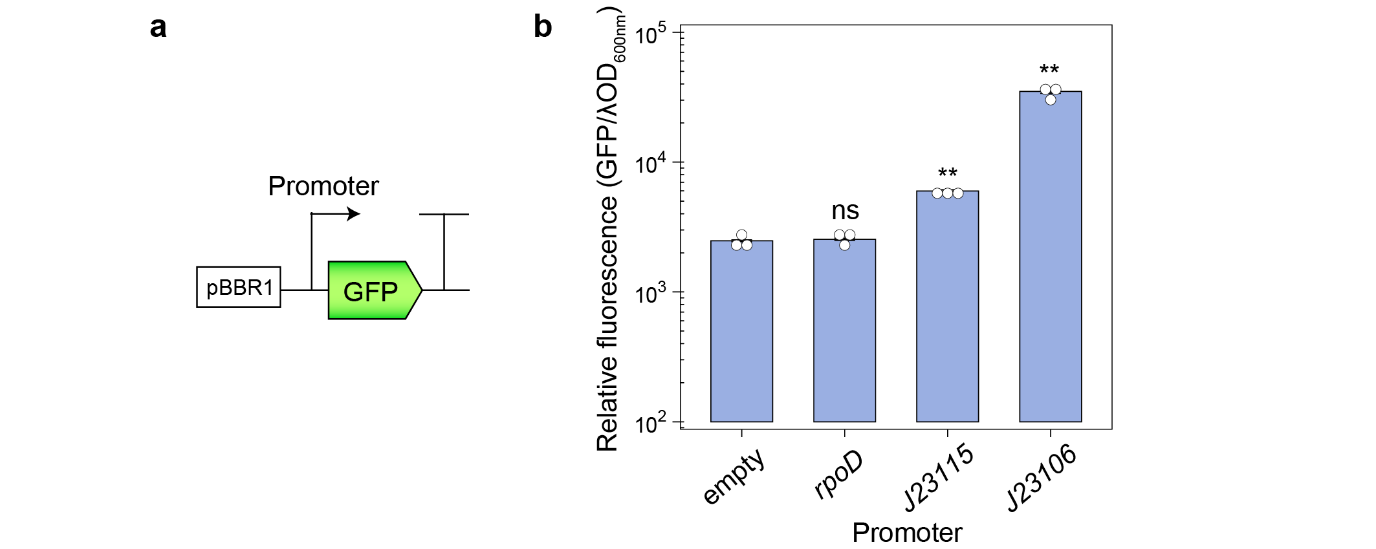
**

**Figure A1. Promoter strengths in *Rlv*3841.** Bacteria were induced for 24-h prior to measurement. Error bars represent one SEM. Independent two-tailed students t-tests using the empty vector control treatment as a reference group were used to compare means. Not significant (ns P > 0.05), **P < 0.01.

**
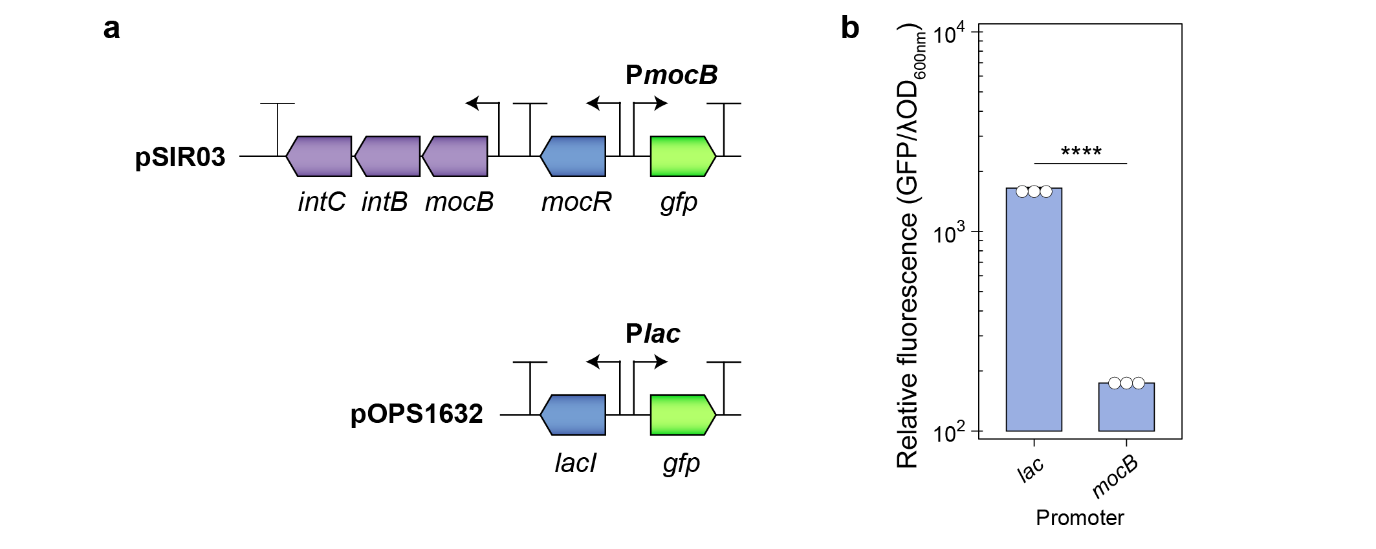
**

**Figure A2. Promoter strengths in CL150.** Bacteria were cultured for 24-h prior to measurement. Error bars represent one SEM. An independent two-tailed students t-test was used to compare means. ****P < 0.01.

**Table A1. Bacterial strains used in this study**

| **Strain** | **Description** | **Reference** |
| --- | --- | --- |
| *Rhizobium leguminosarum* bv. v*iceae (Rlv)3841* | Spontaneous Sm^R^ mutant of strain *R. leguminosarum* 300, symbiont of pea | (1) |
| *Rlv*3841-GFP | *Rlv*3841 carrying J23104::RStd::GFP cassette integrated in single copy into *glmS* with mini-Tn*7* | This study |
| *Rlv*A1350 | *Rlv*3841 *nodD::*Tn*5*-Km, does not nodulate pea | Allan Downie |
| *Rlv*A1350-GFP | *Rlv*A1350 carrying J23104::RStd::GFP cassette integrated in single copy into *glmS* with mini-Tn*7* | This study |
| *Sinorhizobium meliloti* CL150 | *Sm* 2011 with *pstC* and *ecfR1* corrected | (2) |
| *Sm* MB1002 | CL150*ΔnodD1D2D3* | (3) |
| *Sm* MB1003 | CL150*ΔnodD1D2D3 nodC::lacZ* | (3) |
| *E. coli* ST18 | Diparental conjugation strain S17 *λpirΔhemA* | (4) |

**References**

1. Johnston AW, Beringer JE. 1975. Identification of the *Rhizobium* strains in pea root nodules using genetic markers. Journal of General Microbiology 87:343-50.

2. Schlüter J-P, Reinkensmeier J, Barnett MJ, Lang C, Krol E, Giegerich R, Long SR, Becker A. 2013. Global mapping of transcription start sites and promoter motifs in the symbiotic α-proteobacterium *Sinorhizobium meliloti* 1021. BMC Genomics 14:156.

3. Barnett MJ, Long SR. 2015. The *Sinorhizobium meliloti* SyrM regulon: effects on global gene expression are mediated by *syrA* and *nodD3*. J Bacteriol 197:1792-806.

4. Thoma S, Schobert M. 2009. An improved *Escherichia coli* donor strain for diparental mating. FEMS Microbiol Lett 294:127-32.

**Table A2. Plasmids used in this study**

| **Plasmid** | **Replicon** | **Antibiotic resistance** | **Description** | **Ref** |
| --- | --- | --- | --- | --- |
| pBBR-MCS2 | pBBR | Kan | Broad host-range cloning plasmid | (1) |
| pIJ11268 | RK2 | Tet | Stable broad-host-range cloning plasmid with promoterless *luxCDABE* | (2) |
| pLMB712 | RK2 | Tet | pIJ11268 carrying P*nodA_Rlv_* promoter fused to *luxCDABE* | (3) |
| pLMB792 | RK2 | Tet | pIJ11268 carrying P*nodA_Sm_* promoter fused to *luxCDABE* | Alison East |
| pOGG024 | pBBR | Gent | Broad host-range Lv1 golden-gate destination vector | (4) |
| pOGG031 | ColE1 | Spec | pL0M-PU Plac Level 0 golden-gate promoter/RBS module | (4) |
| pOGG037 | ColE1 | Spec | pL0M SC *GFP* Level 0 golden-gate SC module | (4) |
| pOGG059 | ColE1 | Spec | pL1M-ELE2 End linker fragment to close level 2 golden-gate assemblies | Bea Jorrin |
| pOGG072 | ColE1 | Spec | pL0V-SC Level 0 golden-gate SC destination vector | (5) |
| pOGG093 | RK2 | Tet | Stable broad-host-range golden-gate destination vector, very low copy number | (4) |
| pOGG096 | RK2 | Kan | Broad host-range Lv2 golden-gate destination vector | (4) |
| pOGG121 | ColE1 | Spec | pL0M-P PJ23106 Level 0 golden-gate promoter module | Kyle Grant |
| pOGG122 | ColE1 | Spec | pL0M-P PJ23115 Level 0 golden-gate promoter module | Kyle Grant |
| pOGG143 | ColE1 | Spec | pL0M-U [RStd] Level 0 golden-gate RBS module | (6) |
| pOGG157 | ColE1 | Spec | pL0M-T DT16 Level 0 golden-gate terminator module | (6) |
| pOGG162 | ColE1 | Spec | pL0M-T F6S Level 0 golden-gate terminator module | Kyle Grant |
| pOGG250 | RK2 | Tet | Broad host-range Lv1 golden-gate destination vector with *lacI* | This study |
| pOGG262 | ColE1 | Sp | pL0M-SC *nodD_FI_* Level 0 golden-gate CDS module | This study |
| pOGG294 | ColE1 | Sp | pL0M-SC *nodD3* Level 0 golden-gate CDS module | This study |
| pOGG324 | ColE1 | Spec | pL0M-SC *lacI* Level 0 golden-gate CDS module | (7) |
| pOGG331 | RK2 | Carb | pL1M-R1 P*lac*::[RStd]::GFP::DT16 module | This study |
| pOGG367 | RK2 | Carb | pL1M-F2 P*lacIq*::lacI::F6S module | This study |
| pOPS0468 | pBBR1 | Gent | pOGG024 with PJ23106::[RStd]::GFP transcriptional fusion | Beatrice Jorrin |
| pOPS0743 | pBR322 | Gent, Carb | Mini-Tn7 delivery vector carrying PJ23104::[RStd}::GFP | Beatrice Jorrin |
| pOPS1026 | RK2 | Tet | pOGG250 with IPTG-inducible P*lac*::[Rstd]::*nodD3_Sm_* | This study |
| pOPS1090 | RK2 | Tet | pSIR03 carrying *nodD_FI_* and GFP downstream of P*mocB* | This study |
| pOPS1091 | RK2 | Tet | pSIR03 carrying *nodD3_Sm_* and GFP downstream of P*mocB* | This study |
| pOPS1094 | pBBR | Kan | pBBR-MCS2 carrying P*nodD-nodD_Rlv_* | This study |
| pOPS1143 | pBBR1 | Gent | pOGG024 with PJ23115::[RStd]::GFP transcriptional fusion | This study |
| pOPS1330 | pBBR | Gent | pOGG024 with *nodD* and native promoter | This study |
| pOPS1331 | pBBR | Gent | pOGG024 with *nodD_FI_* and native promoter | This study |
| pOPS1368 | pBBR | Gent | pOGG024 with *nodD_FI_* and Plac promoter | This study |
| pOPS1538 | RK2 | Tet | pOGG093 with PJ23106::[Rstd]:: *nodD_FI_* | This study |
| pOPS1632 | RK2 | Kan | pOGG096 with IPTG-inducible Plac::[RStd]::GFP transcriptional fusion | This study |
| pOPS1951 | pBBR1 | Gent | pOGG024 with P*rpoD*::[RStd] GFP transcriptional fusion | This study |
| pOPS1955 | pBBR1 | Gent | pOGG024 with P*empty*::[RStd] GFP transcriptional fusion | (8) |
| pOPS2023 | RK2 | Tet | pOGG093 with PJ23115::[Rstd]::*nodD_FI_* | This study |
| pOPS2024 | RK2 | Tet | pOGG093 with P*rpoD*::[Rstd]:: *nodD_FI_* | This study |
| pSIR03 | RK2 | Tc | Rhizopine-inducible P*mocB* GFP | (7) |
| pSRKKm | pBBR | Kan | Broad host-range cloning plasmid with IPTG-derepressible P*lac* promoter | (9) |
| pTNS3 | R6K | Carb | Tn7 transposase helper plasmid | (10) |

**References**

1. Kovach ME, Elzer PH, Hill DS, Robertson GT, Farris MA, Roop RM, 2nd, Peterson KM. 1995. Four new derivatives of the broad-host-range cloning vector pBBR1MCS, carrying different antibiotic-resistance cassettes. Gene 166:175-6.

2. Frederix M, Edwards A, Swiderska A, Stanger A, Karunakaran R, Williams A, Abbruscato P, Sanchez-Contreras M, Poole PS, Downie JA. 2014. Mutation of praR in *Rhizobium leguminosarum* enhances root biofilms, improving nodulation competitiveness by increased expression of attachment proteins. Mol Microbiol 93:464-78.

3. Pini F, East AK, Appia-Ayme C, Tomek J, Karunakaran R, Mendoza-Suárez M, Edwards A, Terpolilli JJ, Roworth J, Downie JA, Poole PS. 2017. Bacterial biosensors for in vivo spatiotemporal mapping of root secretion. Plant Physiol 174:1289.

4. Geddes BA, Mendoza-Suárez MA, Poole PS. 2019. A bacterial expression vector archive (BEVA) for flexible modular assembly of golden gate-compatible vectors. Frontiers in Microbiology 9:3345.

5. Weber E, Engler C, Gruetzner R, Werner S, Marillonnet S. 2011. A modular cloning system for standardized assembly of multigene constructs. PLoS One 6:e16765.

6. Grant K. 2019. Engineering rhizobacteria as synthetic biology chassis. PhD. University of Oxford.

7. Haskett TL, Karunakaran R, Bueno Batista M, Dixon R, Poole PS. 2022. Control of nitrogen fixation and ammonia excretion in *Azorhizobium caulinodans*. PLoS Genet 18:e1010276.

8. Haskett TL, Geddes BA, Paramasivan P, Green P, Chitnavis S, Mendes MD, Jorrín B, Knights HE, Bastholm TR, Ramsay JP, Oldroyd GED, Poole PS. 2023. Rhizopine biosensors for plant-dependent control of bacterial gene expression. Environ Microbiol 25:383-396.

9. Khan SR, Gaines J, Roop RM, 2nd, Farrand SK. 2008. Broad-host-range expression vectors with tightly regulated promoters and their use to examine the influence of TraR and TraM expression on Ti plasmid quorum sensing. Appl Environ Microbiol 74:5053-5062.

10. Choi KH, Schweizer HP. 2006. mini-Tn7 insertion in bacteria with single attTn7 sites: example *Pseudomonas aeruginosa*. Nature Protocols 1:153-61.
